# Quality of life, fatigue, and muscle strength in women with breast cancer undergoing chemotherapy or hormonal therapy: a case-control study

**DOI:** 10.3389/fonc.2025.1553009

**Published:** 2025-09-29

**Authors:** Naiany P. Silva, Vitor A. Marques, João B. Ferreira-Junior, Ruffo de Freitas-Junior, Carlos A. Vieira

**Affiliations:** ^1^ School of Physical Education and Dance, Federal University of Goiás, Goiânia, Brazil; ^2^ Postgraduate Program in Health Sciences, Federal University of Goiás, Goiânia, Brazil; ^3^ Postgraduate Program in Health Sciences at the Federal University of Goias, Goiania, Brazil; ^4^ Federal Institute of Southeast Minas Gerais, Rio Pomba, Brazil; ^5^ Advanced Center for Diagnosis of Breast Cancer [Advanced Breast Diagnostic Center/Clinics Hospital/Federal University of Goias/Brazilian Hospital Services Company (CORA/HC/UFG/EBSERH)], Clinical Hospital, Federal University of Goiás, Quirinópolis, Brazil

**Keywords:** cancer, psychobiological profile, isometric strength, treatment and mental health, physical exercise

## Abstract

**Background:**

Breast cancer treatments negatively affect women’s physical and emotional well-being due to adverse effects. This study compared the quality of life, fatigue levels, and muscle strength of women with breast cancer undergoing chemotherapy or hormonal therapy with a control group of healthy women.

**Methods:**

A case-control study was conducted including women aged 45 to 65 years diagnosed with breast cancer between October 2021 and August 2022 at the Hospital das Clínicas de Goiânia, Brazil. Approximately one age-matched control was selected from the general population for every 2.2 cases. Quality of life was assessed using the EORTC QLQ-BR23, fatigue with the FACT B + 4, and muscle strength through handgrip tests.

**Results:**

Ninety-five participants were included (65 cases, 30 controls). Among cases, 40% (n = 26) were undergoing chemotherapy and 60% (n = 39) hormonal therapy. Quality-of-life domains such as general symptoms, side effects, and arm and chest symptoms were worse among treated cases compared to controls (p < 0.01 for all). No significant differences were observed between treatment groups (p > 0.05). Fatigue levels were higher among cases compared to controls in domains such as physical and social well-being, general fatigue, cancer- related fatigue, and breast cancer-related fatigue (p < 0.01, p = 0.05, p < 0.01, p < 0.01, p < 0.01, respectively), with no significant differences between treatment groups (p > 0.05). Handgrip strength was lower in cases treated with hormonal therapy on the right side compared to controls (-5.0; 95% CI: -7.69 to -2.31; effect size: -0.49) which represents a moderate magnitude effect. On the left side, both treatment groups showed reduced strength compared to controls (-5.1; 95% CI: -7.99 to -2.21; -5.9; 95% CI: -8.51 to -3.29; effect sizes: -0.93 and -1.04, respectively) indicating effects of large magnitude, which may suggest possible clinical relevance. No significant differences were observed between treatment groups (p > 0.05).

**Conclusion:**

Differences in quality of life, fatigue, and muscle strength were observed between women undergoing treatment and those who were healthy, with no distinction between types of treatment.

## 1 Introduction

Breast cancer remains an important public health problem, although there have been advances in screening and treatment. In 2022, an estimated 2.3 million new cases were diagnosed, making breast cancer the most common cancer among women worldwide (1). With more than 650.000 deaths annually, breast cancer is the fifth most common cause of cancer death worldwide (1). Despite the high incidence of breast cancer deaths, women are increasingly diagnosed with the disease at an early stage, which offers them more treatment options and increases survival rates (2).

Chemotherapy and hormone therapy are effective treatments for increasing survival in breast cancer patients (3), but they can also cause adverse effects, such as loss of muscle strength (4). These treatments can induce muscle atrophy, reduce protein synthesis, and promote inflammatory responses (5). These musculoskeletal changes compromise patients’ physical function and recovery, placing them at greater risk of mortality (4). Studies report that women with breast cancer are 30% to 40% more likely to report an inability to perform daily activities that require physical strength compared to women without a history of breast cancer (5). These effects, in turn, can negatively impact activities of daily living, social interaction, and health-related quality of life (6).

In addition to muscle changes, fatigue is one of the most prevalent symptoms reported by women during treatment (7). Previous studies have shown that about 90% of women with breast cancer experience fatigue during chemotherapy (7, 8), and this effect can persist for years after treatment is completed (9, 10). Although studies on the side effects of treatments provide suggestive data, the consequences of chemotherapy and hormone therapy still constitute a significant problem that requires further investigation. Due to limited data, it is still not possible to accurately determine which treatment is more strongly associated with worse outcomes in the lives of these women.

We aimed to investigate and compare the quality of life, fatigue levels, and muscle strength of women with breast cancer treated with chemotherapy and hormone therapy in relation to a control group of apparently healthy women.

## 2 Methods

A case-control study was conducted and reported according to the Strengthening the Reporting of Observational Studies in Epidemiology (STROBE) guidelines. Cases consisted of women with a confirmed diagnosis of breast cancer, registered and receiving care at the Hospital das Clínicas de Goiânia, Goiás, Brazil. Women with breast cancer were included consecutively, considering all those who sought treatment between October 2021 and August 2022 and met the eligibility criteria. Approximately one age-matched control group was selected from the general population of Goiânia for every 2.2 cases. This ratio reflects the exploratory nature of the study and the use of consecutive sampling, which aimed to include all eligible women with breast cancer during the data collection period. Case inclusion was prioritized to ensure greater clinical representation of the target population. Control group selection, in turn, was limited by the availability of matched individuals without a history of cancer during the same period.

The matching process aimed to ensure similar age distributions between the case and control groups, as evidenced by the means, standard deviations, medians, and interquartile ranges presented in **Table 1**. Controls were also enrolled consecutively, including all volunteers who responded to the recruitment call and met the eligibility criteria.The study was approved by the Research Ethics Committee of the Federal University of Goiás (CAAE: 50717115.4.0000.5083) and by the Research Ethics Committee of the Hospital das Clínicas (CAAE: 50717115.4.3001.5078), in accordance with Resolution No. 466/2012 of the National Health Council for research involving human subjects. Data collection began only after full ethics approval was obtained.

**Table 1:** Sociodemographic characteristics of the participating cases and controls (n=95).

Characteristics	Cases (n = 65)	Controls (n = 30)	*p-* value
Age (y), median (Q1; Q3)^a^	54 (46; 60)	53 (46; 56)	0.511
Weight (kg), mean (SD)^b^	69 (10.1)	70 (12.7)	0.711
Height (cm), median (Q1; Q3)	1.6 (1.5; 1.6)	1.6 (1.5; 1.6)	0.975
BMI (kg/m²), median (Q1; Q3)	27 (25; 29)	27 (23; 31)	0.904
Years of schooling, years, n (%)^c^			
≥ 8	34 (52)	23 (76)	
≤ 8	31 (47)	7 (23)	
Type of treatment, n (%)			
Chemotherapy	26 (40)		
Hormone therapy	39 (60)		
Staging, n (%)			
I	10 (15)		
II	35 (53)		
III	20 (30)		
Lymphedema, n (%)			
Yes	27 (41)		
No	38 (58)		
Type of Surgery, n (%)			
Mastectomy	27 (41)		
Quadrantectomy	38 (58)		
Type of chemotherapy, n (%)			
Adjuvant	38 (58)		
Neoadjuvant	27 (41)		

^a^Results are presented as median (Q1; Q3) or ^b^means and standard deviation (SD). ^c^Categorical variables are expressed as number (%). Y, years; BMI, body mass index.

### 2.1 Participants

Patients were eligible for the study if they had histologically confirmed stage I to III primary breast cancer and were undergoing neoadjuvant or adjuvant chemotherapy and hormonal therapy, including the use of tamoxifen and aromatase inhibitors. Healthy women with no self-reported history of breast cancer or any other type of cancer were allocated to the control group and underwent the same assessment protocol. These participants were originally recruited through public announcements about the study and informational sessions regarding the research. In addition, all participants in both groups were required to be postmenopausal, between 45 and 65 years of age, and not to have participated in any regular physical exercise program in the six months prior to data collection. For the purposes of this study, a regular physical exercise program was defined as participation in structured training sessions at least twice per week.

Only data from women who signed the Informed Consent Form were included in the analysis. The document was completed by the participants themselves during interviews with trained researchers, during which all information about the study was clearly explained and any questions were clarified. The form was signed in duplicate, with one copy retained by the participant and the other kept on file by the research team. Women with severe psychiatric or cognitive impairments that could hinder understanding of the assessment instruments or consent, or severe orthopedic limitations that could compromise the performance of the study’s strength protocol, were excluded. Individuals identified as being at potential risk of psychological distress due to participation were also considered ineligible for the study.

### 2.2 Variables

We measure quality of life, fatigue and handgrip strength. In addition, specific questions were asked about the general state of health, addressing conditions such as hypertension, diabetes, cardiovascular disease and lymphedema. Questions about sociodemographic variables such as marital status, socioeconomic status, and ethnicity were also included. In addition, anthropometric data, such as height (in meters) and weight (in kilograms) of the volunteers, were recorded to calculate the body mass index (weight/height*height). Data on physical activity was also collected.

### 2.3 Procedures experimental

All cancer patients and healthy women received an explanation of the study from the researchers and, when they agreed to participate, they signed the informed consent form. The volunteers filled out the questionnaires related to the study variables during a single meeting with the researchers, where an interview was conducted to assist in the completion and offered support to clarify any doubts about interpretation.

### 2.4 Evaluation of volunteers

Quality of life was assessed using the European Organization for Research and Treatment of Cancer Quality of Life Questionnaire (EORTC – BR 23). This questionnaire contains 23 questions and presents a Likert scale with variation for four answers: 1- no, 2-little, 3- moderately and 4- very much. Questions 31 to 38 and 47 to 53 are associated with the Symptom Scale, while questions 39 to 43 are related to the Functionality Scale. Final scores are calculated independently for each scale, ranging from 0 to 100. Higher scores indicate better quality of life on the functioning scale, while higher scores on the symptom scale represents poorer quality of life. This questionnaire was translated and cross- culturally adapted to Brazilian Portuguese and has a Cronbach’s alpha coefficient between 0.46 and 0.94 (internal) for the different scales (11).

Women’s fatigue was assessed using the Functional Assessment of Cancer Therapy – Breast Cancer + Arm Scale FACT B + 4. This questionnaire consists of 37 questions covering the following domains: 1) physical well-being; 2) social and family well-being; 3) emotional well-being; 4) functional well-being; and 5) breast cancer subscale. The TOI score – Trial Outcomes Index composed of 23 items is a combination of the following subscales: 1) physical well-being; 2) functional well-being and 3) breast cancer subscale. The TOI allows exploring the influence of breast cancer on physical and social aspects. Scores vary according to the scales. The physical and social well-being scales have a score from 0 to 24; functional well-being from 0 to 28; breast cancer subscale from 0 to 36 and the subscale in arms from 0 to 20. The score is calculated independently for each scale by adding the score for each question. The final scores range from 0 to 164 points, with higher scores in each domain representing lower symptoms of fatigue, while lower scores are associated with higher symptoms of fatigue. The Cronbach score of this questionnaire is 0.88, good reproducibility with a correlation coefficient between moments 1 and 2 of 0.97 (12).

Muscle strength was assessed using the handgrip strength test, in kilograms, using a digital dynamometer. The handgrip test, used to quantify the muscle strength of the hand and forearm, served as an indicator of overall muscle function. To perform the procedure, the volunteers were instructed to sit in a chair without armrest, with the shoulders abducted and in neutral rotation. The elbow remained flexed at 90 degrees, with the forearm in a neutral position and the wrist ranging from 0 to 30 degrees of extension, according to the recommendations of the American Society of Hand Therapists (13). The tests consisted of five attempts, each with 3 to 5 seconds of maximal voluntary contraction, under verbal stimulus, alternating between the right and left sides, with a 1-minute rest interval between attempts (13). The highest value obtained among the measurements was used for the analysis. **Table 2** presents each variable, type of variable (e.g., continuous, categorical) and the instruments used.

**Table 2:** Description of the variables included in the study.

Variable	Type of variable	Instruments
Age	Continuos	Anamnesis
Body Mass	Continuos	Anamnesis
Body Mass Index	Continuos	Anamnesis
School of Age	Categorical	Anamnesis
Staging	Categorical	Anamnesis
Lymphedema	Dichotomous	Anamnesis
Type of Surgery	Dichotomous	Anamnesis
Type of Chemotherapy	Dichotomous	Anamnesis
Quality of Life	Continuos	EORTC-BR 23
Fatigue	Continuos	FACT B+4
Handgrip Test	Continuos	Handgrip Test

EORTC-BR 23, European Organization for Research and Treatment of Cancer Quality of Life Questionnaire; FACT B+4, Functional Assessment of Cancer Therapy – Breast Cancer + Arm Scale.

### 2.5 Sample size

A formal sample size calculation was not conducted for this exploratory study. Given its preliminary nature, the sample size was determined based on the number of eligible participants who agreed to participate during the data collection period. The use of consecutive sampling allowed an initial comparison of quality of life, fatigue levels, and handgrip strength between women with breast cancer and health controls. While we recognize that this sampling approach limits generalization of the results to the broader population of women with breast cancer, the data generated can serve as a basis for estimating sample size in future studies.

### 2.6 Statistical methods

The normality of the data was tested using the Shapiro-Wilk test and the homogeneity was evaluated using the Levene test. The variables weight, handgrip strength, functional scale, functional scale linear transfer, and functional well-being showed normal distribution and are expressed as mean ± standard deviation (SD). For handgrip strength, the mean difference between the hormone and chemotherapy treatment groups in relation to the control group was also calculated, with their respective confidence intervals (95% CI) and Cohen’s d effect size was calculated, in order to identify the clinical relevance of the observed differences. The d-values obtained were used to define the effect of chemotherapy and hormonal treatment as trivial (d < 0.2), small (0.2 ≤ d < 0.5), medium (0.5 ≤ d< 0.8) and large (d ≥ 0.8). The variables age, height, body mass index (BMI), symptom scale, symptom scale linear transfer, side effects, hair loss, arm symptoms, chest symptoms, body image, future prospects, sexual function, sexual satislevels, Theical well-being, social well-being, emotional well-being, fatigue related to breast cancer symptoms, total fatigue related to breast cancer, general fatigue levels, the total level of breast cancer-related fatigue was not normally distributed and is presented as median and interquartile range (25%, 75%).

A one-factor ANOVA was performed to compare the data of the variables that presented normal distribution between the groups. In case of significant difference, Tukey’s *post hoc* test was performed. The variable functional well-being did not respect the assumptions of homogeneity, and the Games-Howell *post hoc* test was used. The non-parametric Kruskal-Wallis’s test was performed to compare the data of the variables that did not present normal distribution between the groups. The level of significance adopted was p< 0.05. All analyses were performed using the jamovi project software (2021 version 1.6). The statistical analysis was conducted by a researcher who remained blind to the allocation of participants in the groups in order to reduce possible biases in the interpretation of the results.

## 3 Results

**Figure 1:**
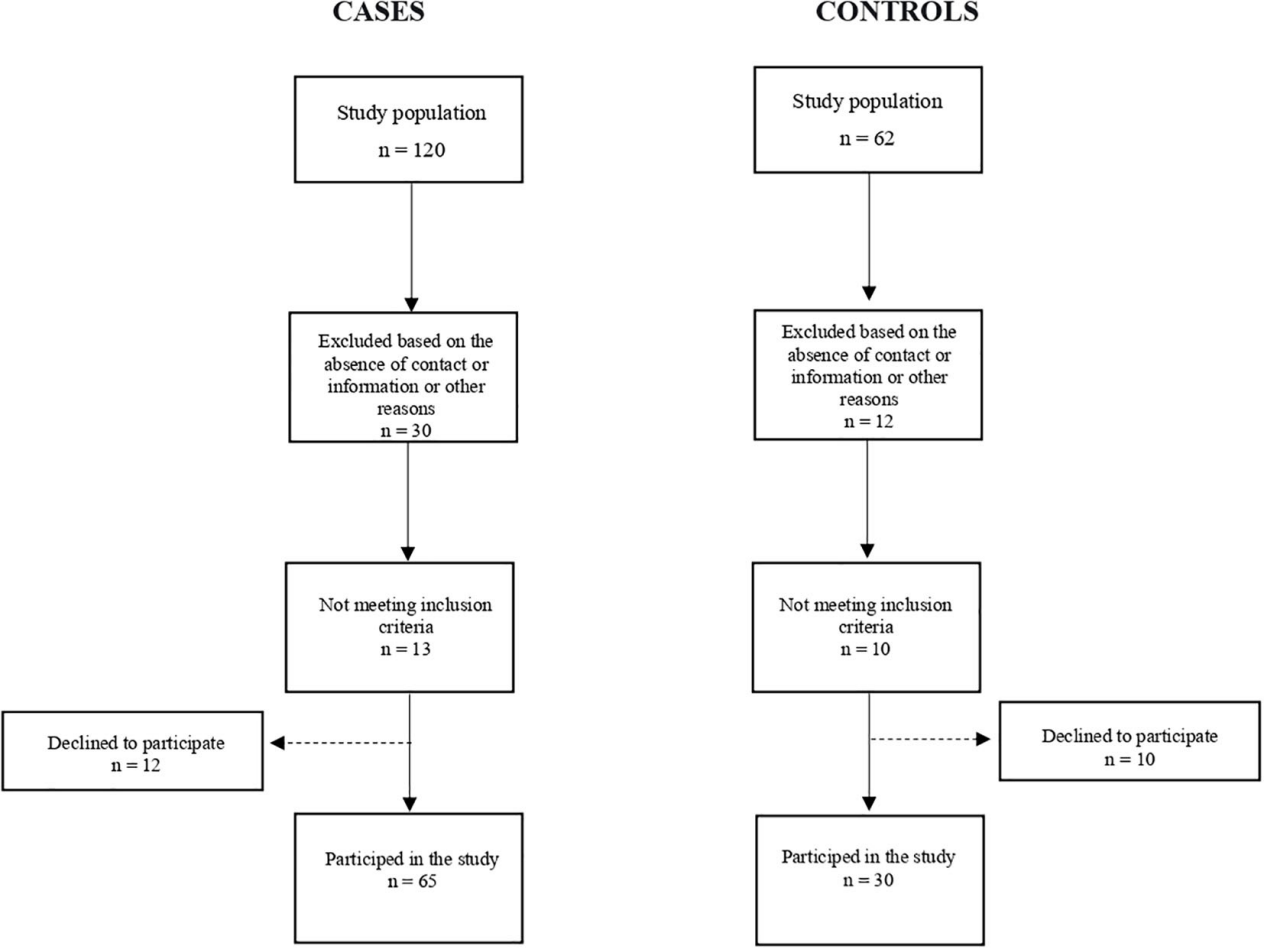
Flowchart describing the inclusion of study participants.

Participants were recruited between October 2021 and August 2022. The study population consisted of 95 individuals (**Figure 1**). At the time of the research, 65 eligible cases with breast cancer (68.4%) and 30 controls (31.5%) were selected. The participating cases and controls were generally similar with respect to sociodemographic characteristics (**Table 1**). Most of the study participants were overweight, had more than 8 years of schooling, and the average age was 54 years. Among the cases, about 40% were undergoing chemotherapy treatment and 60% were on hormone therapy, most had stage II breast cancer (36.8%) and 21.4% had lymphedema. Approximately 28.4% of the cases underwent mastectomy and 40% underwent breast-conserving surgery (quadrantectomy). The prevalence of adjuvant chemotherapy was approximately 40%, while neoadjuvant chemotherapy was 28.4% among cases.

Patients allocated to the chemotherapy and hormone therapy group had a higher presence and intensity of physical and psychological symptoms (general symptoms – 31.87, IQ – 14.67; 48.0 and 22.33, IQ – 10.0; 38.67 respectively), side effects (31.00, IQ – 15.33; 60.67 and 28.67, IQ – 14.33; 52.33 respectively), arm symptoms (22.33, IQ – 11.0; 33.33 and 22.33, IQ – 0.0; 55.67 respectively) and chest symptoms (16.67, IQ - 0.0; 41.67 and 16.67, IQ – 5.33; 33.33 respectively) compared to controls (**Table 3**). The chemotherapy group had a better perception of body image and future perspectives compared to the control group (33.33, IQ – 25.0; 48.0 and 33.33, IQ – 0.0; 100.0 respectively). There was no statistically significant difference between the groups for sexual function and sexual satisfaction. Regarding hair loss, although there was an effect of the treatment (p = 0.03), the comparisons between cases and controls did not reveal statistically significant differences in scores (p> 0.05).

**Table 3:** Median (IQ) of quality-of-life scores between cases and controls.

Variable	Groups			*p-*value
	Cases TQ (n = 26)	Cases TH (n = 39)	Con (n = 30)	
General symptoms (0- 100)	31.87 (14.67; 48.0) *	22.33 (10.0; 38.67) *	85.33 (61.2; 90.9)	<0.01
Side Effects (0- 100)	31.00 (15.33; 60.67) *	28.67 (14.33; 52.33) *	4.67 (0.0; 14.33)	<0.01
Hair loss (0- 100)	0.00 (0.0; 100.0)	0.00 (0.0; 0.0)	0.00 (0.0; 0.0)	0.03
Symptoms in the arm (0- 100)	22.33 (11.0; 33.33) *	22.33 (0.0; 55.67) *	0.0 (0.0; 0.0)	<0.01
Chest symptoms (0- 100)	16.67 (0.0; 41.67) *	16.67 (5.33; 33.33) *	0.0 (0.0; 0.0)	<0.01
Body Image (0- 100)	33.33 (25.0; 48.0)	25.00 (0.0; 66.67) *	0.0 (0.0; 16.67)	<0.01
Future Outlook (0- 100)	33.33 (0.0; 100.0)	33.33 (0.0; 66.67) *	0.0 (0.0; 33.33)	0.04
Sexual function (0- 100)	8.33 (0.0; 33.33)	33.33 (16.67; 66.67)	50.0 (0.0; 83.33)	0.02
Sexual satisfaction (0- 100)	66.67 (0.0; 100.0)	33.33 (0.0; 66.67)	66.67 (0.0; 100.0	0.06

*Significant difference for the control group (p<0.05). TQ Cases, Chemotherapy Cases; TH Cases, Hormone Therapy Cases; Con, Control Group.

Both cases undergoing chemotherapy and hormone therapy had higher fatigue rates compared to controls in the domains of physical well-being (21.0 IQ – 16.3; 26.0 and 22.0 IQ – 19.0; 25.0 respectively), social well-being (18.0 IQ – 14.3; 21.8 and 18.0 IQ – 15.0; 20.0 respectively), general fatigue (*Total Outcomes Index*) (59.0 IQ – 53.0; 67.8 and 66.0 IQ – 54.0; 77.0 respectively), general cancer-related fatigue (Fact G) (74.0 IQ – 69.0; 80.5 and 76.0 IQ – 64.0; 85.5) and breast cancer-related fatigue symptoms (Fact B) (95.0 IQ – 88.5; 107.0 and 101 IQ – 88.0; 118.0 respectively) (**Table 4**). For the functional well-being domain, the cases had lower fatigue rates compared to the controls (16.0± 5.08 and 20.9 ± 3.55, respectively). Only cases on hormone therapy recorded less fatigue related to breast cancer symptoms compared to controls (34.5 IQ – 30.3; 37.0). There was a higher rate of emotional fatigue for the cases undergoing chemotherapy when compared to the controls (18.0 IQ –16.0; 22.0). No differences were observed between the groups for other comparisons (p> 0.05).

**Table 4:** Median (IQ), mean (SD) of fatigue scores between cases and controls.

Variable	Groups			*p-*value
	Cases TQ (n = 26)	Cases TH (n = 39)	Con (n = 30)	
Physical Well-being (0- 24)	21.0 (16.3; 26.0) *	22.0 (19.0; 25.0) *	27.0 (25.0; 28.0)	<0.01
Welfare (0- 24)	18.0 (14.3; 21.8) *	18.0 (15.0; 20.0) *	21.0 (19.0; 24.0)	0.05
Emotional well-being (0- 24)	18.0 (16.0; 22.0) *	20.0 (17.5; 22.0)	21.0 (20.0; 23.0)	0.03
Functional well-being (0- 28)	16.0 ± 5.08 *	20.9 ± 3.55*	15.6 ± 6.19	<0.01
Breast Cancer Symptoms (0- 36)	34.5 (30.3; 37.0)	16.67 (5.33; 33.33) *	28.0 (21.5; 32.0)	<0.01
Fact B TOI (0- 92)	66.0 (54.0; 77.0) *	25.00 (0.0; 66.67) *	81.0 (76.3; 87.0)	<0.01
Fact G Total (0- 108)	76.0 (64.0; 85.5) *	33.33 (0.0; 66.67) *	90.0 (82.3; 98.0)	<0.01
Fact B Total (0- 144)	101 (88.0; 118.0) *	33.33 (16.67; 66.67) *	125 (116; 135)	<0.01

*Significant difference for the control group (p<0.05). TQ Cases, Chemotherapy Cases; TH Cases, Hormone Therapy Cases; Con, Control Group. Fact B Toi = overall fatigue index, total Fact G = overall cancer-related fatigue index, total Fact B = breast cancer-related fatigue index.

In the analysis of handgrip strength, the cases treated with hormone therapy showed lower right-hand grip strength than participants without breast cancer (–5.0; 95% CI: –7.69 to –2.31), with an effect size of 0.49 (**Table 5**), which represents a moderate magnitude effect, but not a clinically significant one. Regarding left-hand grip strength, both treatment groups showed lower strength compared to the controls (–5.1; 95% CI: –7.99 to –2.21 and –5.9; 95% CI: –8.51 to –3.29, respectively), with effect sizes of –0.93 and –1.04, respectively, indicating effects of large magnitude, which may suggest possible clinical relevance. There were no differences between the cases undergoing chemotherapy and those receiving hormone therapy (p >0.05).

**Table 5:** Mean (SD) for handgrip strength of each group and mean difference (95% CI) in relation to the control group.

Variable	Groups			Mean difference (95% CI) compared to controls	Effect size	*p-*value
	Cases TQ (n = 26)	Cases TH (n = 39)	Con (n = 30)			
HT right (kgf)	25.8 ± 5.31	23.3 ± 6.79*	28.3 ± 4.58	TQ: - 2.5 (IC: - 5.12 a – 0.12)	-0.49	0.02
				TH: - 5.0 (IC: - 7.69 a – 2.31)	- 0.49	
HT left (kgf)	22.2 ± 6.35*	21.4 ± 6.70*	27.3 ± 4.33	TQ: - 5.10 (IC: - 7.99 a – 2.21)	- 0.93	<0.01
				TH: - 5.90 (IC: - 8.51 a 3.29)	-1.04	

*Significant difference for the control group (p<0.05). HT, handgrip test; TQ cases, chemotherapy cases; TH cases, hormone therapy cases; Con, control group; kg, kilogram force.

## 4 Discussion

Although chemotherapy and hormone therapy decrease the risk of breast cancer recurrence, the side effects resulting from treatment greatly affect quality of life. These effects increase fatigue levels and cause musculoskeletal damage, which reduces muscle strength (14). Our results suggest that individuals with breast cancer have, in most of the domains analyzed, a worse quality of life, higher fatigue rates and lower handgrip strength compared to individuals without breast cancer. Despite identifying the effect of the treatments for the outcomes analyzed, we did not find significant differences between patients treated with chemotherapy and those who received hormonal therapy.

We observed that the cases under treatment had lower quality of life in four of the nine domains evaluated and higher fatigue rates in six of the eight variables associated with fatigue symptoms. In addition, handgrip strength was significantly lower in patients compared to controls, with a mean difference of 5.3 kgf, characterizing a mean effect. Based on this, we are confident that the reduction in handgrip strength is clinically relevant, given that the effect size is like that reported in previous studies that compare the handgrip strength of breast cancer patients undergoing chemotherapy with healthy individuals (14, 15). However, causality to inference is limited because it is a case-control study.

During breast cancer treatment, a marked deterioration in the patient’s quality of life usually occurs, and it is well known that this is related to the side effects of chemotherapy and hormone therapy (16, 17). A study by our research team suggested that chemotherapy between the third and fourth cycle may negatively affect quality of life in three of the eight domains of the Short-Form Healthy Survey (SF-36) (15). One observational study compared the quality of life of women with breast cancer who received chemotherapy with those who did not. The results show that chemotherapy worsens quality of life, especially in the domains of physical functioning and physical role (18). As part of the increased survival in breast cancer patients, hormone therapy has been introduced to prolong disease-free survival (19). However, studies report that quality of life is affected by the prolonged use of adjuvant hormone therapy, especially among patients who received anastrozole, tamoxifen, and exemestane (20). Other side effects categorized by chemotherapy and hormone therapy are related to fatigue. Previous studies have observed increased fatigue in women with breast cancer after adjuvant chemotherapy (21), which has been attributed to the use of taxanes, whose side effect is fatigue and pain (22). Somatic and psychosocial factors are associated with fatigue in patients treated with hormone therapy (23–25). These findings are consistent with the results of the present study, as compared to women with no history of cancer, women treated with chemotherapy and hormone therapy reported that fatigue interfered more with their general activities, physical and social well-being.

The current results also demonstrate a statistically significant and clinically important difference for handgrip strength between cases and controls. Previous research shows that muscle strength is a prognostic factor for breast cancer patients that correlates with both survival and how well patients respond to treatment (26). Low levels of palmar pressure strength are associated with chemotherapy toxicity (27) and musculoskeletal side effects caused by drugs used during hormone therapy, which possibly impair muscle protein renewal (28–30). One limitation of all the studies mentioned (including our own) is that patients are followed for only a single point during the different types of treatment, which limits the conclusions that can be drawn. The lack of prospective follow-up does not provide information on changes in outcomes that may have already occurred at the beginning of treatment or that may occur throughout the treatment phases.

Firstly, this is the first study to investigate quality of life, fatigue levels, and muscle strength in breast cancer patients treated chemotherapy and hormone therapy using a case-control design. We included several strategies to minimize bias, such as using a sample of cases and similar controls on sociodemographic attributes. In addition, the questionnaires used were validated and widely accepted in the scientific literature, and the application of the questionnaires and data analysis were conducted by independent professionals. The use of a comparable control group also strengthens the conclusions.

The limitations of our study included the use of a small, consecutive sample, which undermines external validity and may reduce confidence in drawing meaningful and generalizable conclusions for other populations of women with breast cancer, especially those undergoing different treatment modalities. Furthermore, the outcome assessment was conducted at a single time point, which prevents understanding changes over time. Future studies with longitudinal follow-up may provide a more detailed view of variations in quality of life, fatigue, and muscle strength. Finally, it was not possible to precisely distinguish the proportion of the observed changes in outcomes that could be related to oncological treatment from those that might be associated with the clinical condition of breast cancer itself, which should be considered when interpreting the results.

## 5 Conclusion

We found statistically significant differences in quality of life, levels of fatigue, and muscle strength between women with breast cancer undergoing chemotherapy or hormone therapy compared to healthy women. No statistically significant differences were observed between the treatment groups, suggesting that both modalities may be associated with similar changes in these outcomes. Despite statistical significance, the results did not indicate clinically relevant differences in quality of life and fatigue. On the other hand, the reduction in muscle strength observed in women with breast cancer may represent a clinically relevant difference. These findings highlight the need for complementary strategies that can mitigate the possible adverse consequences associated with cancer treatments, such as structured physical exercise programs, which should be investigated in future studies.

## Data Availability

The original contributions presented in the study are included in the article/supplementary material. Further inquiries can be directed to the corresponding author.
